# Adsorption Performance of Cd(II) by Chitosan-Fe_3_O_4_-Modified Fish Bone Char

**DOI:** 10.3390/ijerph19031260

**Published:** 2022-01-23

**Authors:** Wenhao Yang, Wenwen Luo, Tong Sun, Yingming Xu, Yuebing Sun

**Affiliations:** 1 Key Laboratory of Original Environmental Pollution Prevention and Control, Ministry of Agriculture and Rural Affairs, Tianjin 300191, China; 18865712611@163.com (W.Y.); 17602669062@163.com (W.L.); 17862685017@163.com (T.S.); xuyingming@aepi.org.cn (Y.X.); 2 Tianjin Key Laboratory of Agro-Environment and Agro-Products, Agro-Environmental Protection Institute, Ministry of Agriculture and Rural Affairs, Tianjin 300191, China; 3 College of Resources and Environment, Northeast Agricultural University, Harbin 150030, China

**Keywords:** Cd, fish bone char, Fe_3_O_4_, chitosan, modification, adsorption mechanism

## Abstract

In order to develop a low-cost, fast, and efficient adsorbent, the fish bone charcoal B_600_ prepared at 600 °C was modified by chitosan (Cs) and Fe_3_O_4_ to produce the material Cs-Fe_3_O_4_-B_600_. Results showed that Cs-Fe_3_O_4_-B_600_ had magnetic responsiveness and can achieve solid–liquid separation, macropores disappeared, pore volume and specific surface area are increased, and amino functional groups appear on the surface. The adsorption process of Cd(II) by Cs-Fe_3_O_4_-B_600_ conformed best to the pseudo-second order kinetics model and the Langmuir model, respectively. The behavior over a whole range of adsorption was consistent with chemical adsorption being the rate-controlling step, which is a very fast adsorption process, and the isothermal adsorption is mainly monolayer adsorption, which belongs to favorable adsorption. In addition, the saturated adsorption capacity obtained for the Cs-Fe_3_O_4_-B_600_ to Cd(II) was 64.31 mg·g^−1^, which was 1.7 times than B_600_. The structure and morphology of Cs-Fe_3_O_4_-B_600_ were characterized through SEM-EDS, TEM, FTIR, and XRD, indicating that the main mechanism of Cs-Fe_3_O_4_-B_600_ and Cd(II) is mainly the complexation of amino groups, and it also includes part of the ion exchange between Cd(II) and Fe_3_O_4_. Therefore, Cs-Fe_3_O_4_-B_600_ can be employed as an effective agent for remediation of Cd contaminated water.

## 1. Introduction

Cadmium (Cd) is a highly toxic carcinogen, which enters the human body through the food chain and causes damage to human health [1,2]. Human intake of Cd mainly includes food, smoking, and drinking water. Among them, grains and vegetables are the largest sources of human long-term intake of Cd [3]. Cd in the soil is easily absorbed by plants and transported in their tissues [4]. At present, Cd content of many food crops in China has exceeded the corresponding national food safety standard [3,5]. Wastewater irrigation is an important cause of cadmium pollution in agricultural soil. Therefore, it is very important to remove cadmium in wastewater.

Cd pollution cannot reduce or even eliminate its harmfulness through environmental self-purification but can only achieve the transformation of the occurrence state and the migration of the location. A variety of remediation measures are used for the remediation of Cd-contaminated soils, such as physical and chemical remediation, microbial remediation, and phytoremediation [6]. Currently, the adsorption method has attracted much attention due to its high efficiency, low cost, and non-secondary pollution. Adsorption is considered to be a process in which molecules gather from a fluid to a solid surface. In recent years, the use of low-cost, widely sourced industrial/agricultural/domestic waste or by-products as adsorbents to remove heavy metals in water bodies has received widespread attention [7,8,9,10]. Bone char mainly includes hydroxyapatite (70–76%), a small part of char (9–11%) and carbonate (7–9%) [11]. Several studies have demonstrated that bone char has a good adsorption effect on heavy metals Cu(II), Zn(II), Co(II), Hg(II) [12,13,14]. For example, the research of Wang [15] and Liu [16] showed that bone char also has a good treatment effect on Pb(II) and As(V), and the adsorption capacity can reach 84.75 and 0.335 mg·g^−1^, respectively. In addition, Moreno et al. [17] found that the saturated adsorption capacity of bone char for Mn and Ni can reach 29.56 and 35.44 mg·g^−1^, respectively. Sneddon et al. [18] used column leaching experiments to study the fixation effect of bone char on Pb(II), Zn(II), and Cd(II) in soil, that is, to test the stability of metals in contaminated soil by forming low-soluble metal phosphates. The results show that when bone char:soil = 1:10 (mass ratio), the release of heavy metals is inhibited during the whole process of the experiment, which may involve surface complexation and ion exchange.

The modification of bone meal materials usually refers to the use of impregnation or co-precipitation methods to load target functional groups or target components on the surface of the material. Common composite modifications include metal ion loading modification such as aluminum, iron, lanthanum, sulfhydryl, and other surface group loading modification, graphene modification, γ-Fe_2_O_3_, Fe_3_O_4_, NiFe_2_O_4_, CoFe_2_O_4_, CuFe_2_O_4_, and ZnFe_2_O_4_ particles and other magnetic properties modified [19,20,21,22,23,24]. However, compared with bone meal, bone char materials have well-developed pores and are more suitable as a carrier for loading materials. Therefore, the composite modification of bone meal materials is mostly performed on the bone char obtained by treatment at different temperatures.

Studies have shown that magnetic materials not only easily achieve solid–liquid separation, but also have a good removal effect on heavy metals. For example, functionalized magnetic microspheres NiFe_2_O_4_ can adsorb Cu up to 20.16 mg·g^−1^ [25], FeS-coated iron can adsorb Cr(VI) up to 69.7 mg·g^−1^ [26], FeNi_3_/TiO_2_ material can adsorb Cr(VI) up to 76.335 mg·g^−1^ [27]. Among the above-mentioned magnetic particles, Fe_3_O_4_ is widely used because of its strong superparamagnetism, low toxicity, and easy synthesis [28,29]. The typical synthesis process of nano Fe_3_O_4_ particles mainly includes DC (Direct Current) arc plasma method, thermal decomposition method, co-precipitation method, hydrothermal method, and microemulsion method. Du et al. [30] used the co-precipitation method to prepare Fe_3_O_4_ magnetic biochar and applied it to the treatment of heavy-metal polluted wastewater. The results show that after the magnetic modification, the specific surface area of the adsorbent increases, and the removal rate of Cu and Zn can reach 61.1% and 60.4%, respectively. During the modification process, functional groups such as hydroxyl and carboxyl groups increased [30]. Hu et al. [31] studied the removal of Cd(II) by magnetically modified corn stalk biochar, and the results showed that after the material was magnetically modified, the pH value, specific surface area, and polar oxygen-containing functional groups all increased, resulting in a saturated adsorption capacity. The mechanism of magnetic biochar to remove Cd is ion exchange, surface complexation, electrostatic adsorption, and cation-π interaction. The strong affinity of iron oxide for Cd can enhance the complexation between them [31]. In addition, chitosan is a low cost, biodegradable, and nontoxic biopolymer [32]. Chitosan has a large number of amino functional groups and has strong adsorption capacity for heavy metals in aqueous solution, so it can be widely used in the removal of heavy metal pollutants [33]. However, due to the low specific surface area and limited active sites of chitosan, its adsorption capacity is still insufficient [34]. Chitosan can be introduced on the surface of fish bone char to enhance the adsorption capacity and active sites of fish bone char for heavy metals [34].

However, there are few studies on the effect and mechanism of chitosan and Fe_3_O_4_ composite modified bone char for Cd removal. In this work, we successfully prepared a novel chitosan combined Fe_3_O_4_ modified fish bone char (Cs-Fe_3_O_4_-B_600_), which can be used as a suitable adsorbent for cadmium solution. The objectives of this study were to (1) prepare and characterize the Cs-Fe_3_O_4_-B_600_ and (2) explore the capabilities and mechanisms of Cs-Fe_3_O_4_-B_600_ for adsorption on Cd.

## 2. Materials and Methods

### 2.1. Adsorbent Preparation

#### 2.1.1. Preparation of Fish Bone Char

The fish bone meal used in the experiment was pulverized by a universal pulverizer and passed through a 100-mesh sieve. A part of the sieved fish bone meal was selected, and N_2_ was used as a protective gas to react in a muffle furnace (SLX1100-50, Shanghai Shengli Testing Instrument Co., Ltd., Shanghai, China) at 200, 400, 600, and 800 °C for 3.5 h to obtain fish bone char treated at different temperatures. By measuring the Cd adsorption capacity of fish bone char prepared at different temperatures, we found that fish bone char prepared at 600 °C (B_600_) had the highest adsorption capacity, so B_600_ was selected for subsequent experiments. B_600_ was grind pulverized and passed through a 200-mesh sieve for use. In addition, the basic properties of fish bone meal are as follows. The main component of fish bone meal is Ca_10_(PO_4_)_6_(OH)_2_, the specific surface area is 2.27 m^2^·g^−1^, the pore volume is 0.0035 cm^3^·g^−1^, and the average pore diameter is 4.811 nm.

#### 2.1.2. Preparation of Nano Fe_3_O_4_

The ultrapure water was boiled and then cooled and sealed for later use. After ferrous chloride tetrahydrate (0.0994 g, Shanghai Macklin Biochemical Co., Ltd., Shanghai, China) and ferric chloride hexahydrate (2.7029 g, Shanghai Macklin Biochemical Co., Ltd., Shanghai, China) were dissolved in the above ultrapure water (100 mL) in a three-necked flask, ammonia water with a concentration of 3.5 M was added dropwise, and then vigorously stirred with a Vortex Mixer (QL-866, Haimen Kylin-Bell Lab Instruments Co., Ltd., Haimen, China) to mix well and when the measured pH was 10, addition of ammonia water (about 40 mL) was stopped. In this process, the nitrogen-blowing device (N-EVAP, Organomation, Berlin, MA, USA) was used to pass N_2_ to undertake the reaction under anaerobic conditions. The obtained precipitate was washed with deionized water and dried in a muffle furnace at 70 °C. The above process was repeated several times to obtain a sufficient amount of nano Fe_3_O_4_.

#### 2.1.3. Preparation of Chitosan-Fe_3_O_4_-Modified Fish Bone Char

Chitosan (100 g, Shanghai Macklin Biochemical Co., Ltd., Shanghai, China) was added to absolute ethanol (1000 mL) and stirred vigorously for 2 h to obtain a viscous gum. Bone char (5 g) (B_600_, 200-mesh sieve) and nano Fe_3_O_4_ (2 g) were added to the above viscous gel, and the Vortex Mixer was vigorously stirred for 1 h. The above homogeneous mixture was added dropwise to 500 mL 15% NaOH and 95% absolute ethanol mixture (volume ratio 4:1) with a rubber tip dropper, and continuously stirred to produce chitosan-Fe_3_O_4_-modified fish bone char mixture. After keeping in the solution for 12 h, the precipitate was collected, washed with deionized water to remove surface impurities, dried in a muffle furnace at 70 °C, and sieved for later use. The resulting material was recorded as Cs-Fe_3_O_4_-B_600_. This method refers to Reza’s magnetic modification of bone char [35] and optimizes it on this basis.

### 2.2. Characterization

The magnetic properties of Cs-Fe_3_O_4_-B_600_ were characterized by a Vibrating Sample Magnetometer (Mpms Squid, American quantum design company, San Diego, CA, USA) at room temperature, applying a magnetic field of −20 kOe–20 kOe; the specific surface area and pore volume of Cs-Fe_3_O_4_-B_600_ were measured by a specific surface area analyzer (Quadrasorb Si, Quanta Instruments, Inc., Boynton Beach, FL, USA) using multipoint BET (Method of Brunauer, Emmett and Teller) method and BJH (Method of Barrett, Joyner and Halenda). The multifunctional X-ray diffractometer (Bruker D8 Advance, Bruker, Karlsruhe, Germany) was used to perform XRD (diffraction of x-rays) analysis on Cs-Fe_3_O_4_-B_600_, with a scan range of 10°–90°, a scan rate of 4°·cm^−1^, and a scan step size of 0.02°; Fourier transform infrared spectrometer (Thermo Nicolet 380, Madison, WI, USA) was used for FTIR (Fourier Transform Infrared Spectrometer) analysis of Cs-Fe_3_O_4_-B_600_; The measured wavenumber range was 400–4000 cm^−1^ and the resolution was 1 cm^−1^; Scanning electron microscope (Hitachi SU8200, Hitachi technologies, Tokyo, Japan) and energy dispersive X-ray spectrometer (Hitach, Hitachi technologies, Tokyo, Japan) were used in combination to analyze the sample by SEM-EDS; Transmission electron microscope (JEOL JEM 2010FEF, Jeol, Tokyo, Japan) was used for TEM (Transmission Electron Microscope) analysis of Cs-Fe_3_O_4_-B_600_.

### 2.3. Adsorption

To prepare the standard stock solution, ultrapure water and Cd(NO_3_)_2_·4H_2_O were used to prepare the Cd standard stock solution. In the experiment, ultrapure water was used to dilute the standard stock solution to the required concentration.

To study the adsorption kinetics, Cd(II) solution (25 mL) with an initial pH of 5.40 and an initial concentration of 200 mg·L^−1^ was added to a 50 mL conical flask, and then adsorbent (0.05 g) was added to the Cd(II) solution, under the conditions of 25 °C, 200 r·min^−1^, using a constant temperature culture oscillator (ZHWY-2102C, Shanghai ZHICHENG analytical Instrument Manufacturing Co., Ltd., Shanghai, China) to oscillate for 1, 3, 5, 10, 20, 30, 40, 60, 90, 120, 180, 240, 300, 360, 480, 600, 720, and 1440 min. Finally, the resulting solution was passed through a 0.45 μm water filter membrane, and an atomic absorption spectrophotometer (ZEEnit 700P, Jena Analytical Instrument Co., Ltd., Thuringia, Germany) was used to determine the concentration of Cd(II) in the filtrate. At the same time, the pH of the solution was measured at the above time point. The sample loaded with Cd was washed with ultrapure water and dried at 70 °C and it was recorded as Cs-Fe_3_O_4_-B_600_-Cd.

To study the adsorption isotherm, a series of Cd(II) solutions (25 mL) with an initial pH of 5.40 were added to a 50 mL conical flask, adsorbent (0.05 g) was added to the Cd(II) solution, and then under the conditions of 25 °C and 200 r·min^−1^, the constant-temperature culture oscillator was used to oscillate for 12 h, and the resulting solution was passed through a 0.45 μm water-based filter membrane. An atomic absorption spectrophotometer was used to determine the Cd(II) concentration in the filtrate.

To study the effect of pH on the adsorption performance of Cd(II), Cd(II) solution (25 mL) with an initial concentration of 100 mg·L^−1^ and an initial pH of 3–8 was added to a 50 mL conical flask, and then, adsorbent (0.05 g) was added to the Cd solution. At 25 °C and 200 r·min^−1^, a constant temperature incubation oscillator was used to oscillate for 30 min. Finally, the resulting solution was passed through a 0.45 μm water filter membrane, and an atomic absorption spectrophotometer was used to determine the concentration of Cd(II) in the filtrate.

### 2.4. Statistical Analysis

The adsorption capacity of the adsorbent for Cd(II) was calculated using the following formula:

(1) $$q_{e}=\frac{(C_{0}-C_{t})\times V}{m}$$

where *C*_0_ and *C_t_* represent the initial concentration of Cd(II) in the solution (mg·L^−1^) and the concentration of Cd(II) in the solution at the adsorption time *t* (min) (mg·L^−1^), respectively; *V* is the volume of the solution (L); *m* is the mass of the adsorbent (g).

All detected data were repeated three times, and all treatments were repeated three times independently. The average value was used as the measurement result. The average value was calculated by Microsoft Excel 2010 and Origin 8.6 (OriginLab Corporation, Commonwealth of Massachusetts, America) was used for graphing.

## 3. Results and Discussion

### 3.1. Characterization of Cs-Fe_3_O_4_-B_600_

Fe_3_O_4_ was characterized by XRD as shown in Figure S1. The XRD pattern of Fe_3_O_4_ has obvious diffraction apex and corresponding crystal face at 2θ = 30.091° (220), 35.443° (311), 43.074° (400), 56.964° (511), 62.553° (440). The peak shape of PDF#85-1436 is in good agreement, indicating that its main component is Fe_3_O_4_, which is a pure inverse sine spinel material with a single-phase [36]. In addition, the X-ray diffraction pattern of Fe_3_O_4_ has a sharp diffraction apex, indicating that the self-made Fe_3_O_4_ has high crystallinity [37].

**Figure 1:**
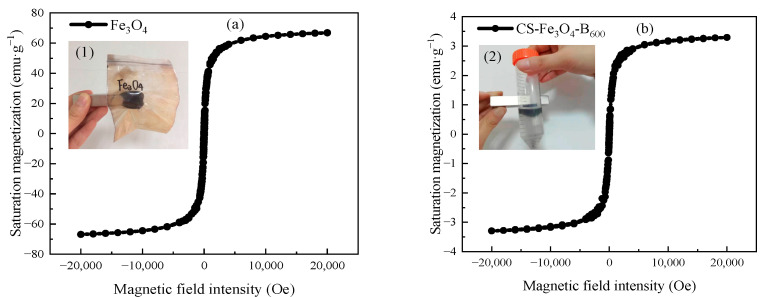
Hysteresis loop of Fe_3_O_4_ (**a**) and Cs-Fe_3_O_4_-B_600_ (**b**). Note: (**a**) shows the magnetism of Fe_3_O_4_ and (**b**) exhibits the magnetic separation of Cs-Fe_3_O_4_-B_600_ with a hand magnet.

The magnetic hysteresis loops of Fe_3_O_4_ and Cs-Fe_3_O_4_-B_600_ are shown in Figure 1. It can be seen that the magnetic hysteresis loops of Fe_3_O_4_ (Figure 1a) and Cs-Fe_3_O_4_-B_600_ (Figure 1b) all pass through the origin, no hysteresis phenomenon occurs, and the coercivity and remanence magnetic are both zero [38]. The magnetic hysteresis loop presents an “S” shape, indicating that Fe_3_O_4_ and Cs-Fe_3_O_4_-B_600_ have superparamagnetism [15]. When the applied external field intensity is 20,000 Oe, the saturation magnetization of Fe_3_O_4_ and Cs-Fe_3_O_4_-B_600_ measured at room temperature is 66.90 and 3.30 emu·g^−1^, respectively. The research of Huang and Tang [39] measured the saturation magnetization of pure iron bulk Fe_3_O_4_ as 88 emu·g^−1^; the saturation magnetization of Fe_3_O_4_ synthesized by the co-precipitation method is 72.1 emu·g^−1^ [40]; the saturation magnetization of Fe_3_O_4_ prepared in a spiral microreactor by co-precipitation method is 53 emu·g^−1^ [15] and the above research results are similar to the measured values in this experiment. The significant decrease in saturation magnetization of Cs-Fe_3_O_4_-B_600_ (as low as 3.30 emu·g^−1^) proves the successful synthesis of composite materials [41]. At the same time, we can see from the embedded picture (2) of Figure 1b that Cs-Fe_3_O_4_-B_600_ can be collected on the surface of the liquid by the magnet, and the liquid becomes clear. That is, although the saturation magnetization of the composite material is significantly lower than that of Fe_3_O_4_, it still has magnetic responsiveness and can achieve solid–liquid separation. The magnetic biochar prepared by Yuan et al. [42] by hydrothermal carbonization has strong ferromagnetism, the coercivity and remanence are 0.0 Oe and 0.0 emu·g^−1^, respectively, and the saturation magnetization reaches 16.7 emu·g^−1^. When the magnet is close to the aqueous solution containing magnetic biochar, the biochar particles are immediately attached to the bottle wall near the magnet, and the water becomes transparent at the same time, which is conducive to the recovery and reuse of biochar as an adsorbent.

**Figure 2:**
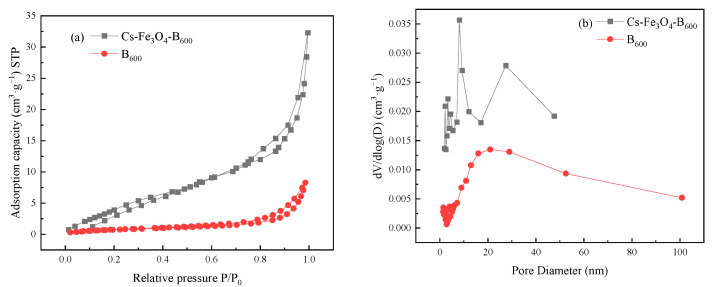
(**a**) N_2_ adsorption–desorption isotherms and (**b**) pore size distribution (BJH) of B_600_ and Cs-Fe_3_O_4_-B_600_. Note: STP means standard temperature and pressure.

The pore structures of B_600_ and Cs-Fe_3_O4-B_600_ were characterized by N_2_ adsorption method. It can be seen from Figure 2a that the N_2_ adsorption–desorption isotherm of B_600_ belongs to a typical type IV curve, and there is an obvious H3 hysteresis loop in the range of P/P_0_ = 0.2–0.9, indicating that the material belongs to mesoporous material. The N_2_ adsorption–desorption isotherms of Cs-Fe_3_O_4_-B_600_ are a typical IV curve, and the obvious H3 hysteresis loop appeared in the range of P/P_0_ = 0.6–0.9. It is generally believed that the H3 hysteresis loop is caused by the slit pores formed by the accumulation of sheet shaped particles [43], and the adsorption is the coagulation capillary effect of the mesopores [44]. According to the IUPAC pore size classification, Figure 2b shows that Cs-Fe_3_O_4_-B_600_ is mainly mesopores (2–50 μm). The pore volume and pore diameter measured by the BJH equation is 0.047 cm^3^·g^−1^, 9.764 nm, and the specific surface area measured by BET is 19.287 m^2^·g^−1^. B_600_ contains a large number of mesopores and macropores. The pore volume, pore diameter, and specific surface area are 0.014 cm^3^·g^−1^, 19.97 nm, and 2.47 m^2^·g^−1^, respectively. By comparison, the composite material has a large pore volume, high specific surface area, and small pore diameter. The disappearance of the macropores of the composite material may be caused by the blockage of the macropores caused by the attachment of chitosan and Fe_3_O_4_ to the surface of the fish bone char. In addition, after modification, the specific surface area of biochar is greatly increased, providing more adsorption sites [45]. After the biochar prepared by Du et al. [46] was modified by Fe_3_O_4_, its specific surface area increased from 117.595 to 145.963 m^2^·g^−1^, and its pore volume increased from 0.05256 to 0.06549 cm^3^·g^−1^. After the rice husk biochar prepared by Zhang et al. [47] was modified with Fe_3_O_4_, its specific surface area and pore volume increased by 99.88 m^2^·g^−1^ and 0.238 cm^3^·g^−1^, respectively; for the Fe_3_O_4_ modified biochar prepared by Wang et al. [48], when the Fe^2+^ concentration increased to 0.4 mol·L^−1^, the specific surface area of the biochar increased significantly from 1.856 to 16.223 m^2^·g^−1^, and the pore volume increased from 0.011 to 0.064 cm^3^·g^−1^. The increases in specific surface area, pore volume, and pore size of the Fe_3_O_4_ modified biochar prepared in the above study are similar to this experimental study.

### 3.2. The Performance of Cd(II) Adsorption on Cs-Fe_3_O_4_-B_600_

#### 3.2.1. Adsorption Kinetic Characteristics

**Figure 3:**
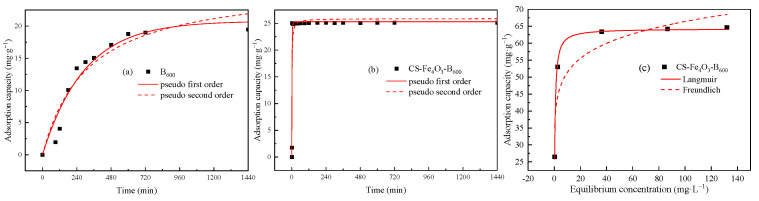
Fitting of kinetics of Cd(II) on B_600_ (**a**) and Cs-Fe_3_O_4_-B_600_ (**b**) and isotherm fitting of Cd(II) on Cs-Fe_3_O_4_-B_600_ (**c**).

Figure 3 shows the changing trend of Cd(II) adsorption capacity of B_600_ and Cs-Fe_3_O_4_-B_600_ with the continuation of the reaction time. We can see that Cs-Fe_3_O_4_-B_600_ more easily to reaches adsorption equilibrium than B_600_, and it only takes 3 min. Li et al. [49] prepared nano-magnetic calcium dihydrogen phosphate and used it for the removal of Cd(II). Studies have shown that the process is a fast adsorption process, and it only takes 1 min to reach the adsorption equilibrium, involving surface adsorption, electrostatic interaction, ion exchange, complexation, and chelation [49]. In addition, the removal of As(V) by magnetite-modified water hyacinth biochar requires 5 min to reach the adsorption equilibrium [44]; the removal of Cd(II) and Pb(II) by Fe_3_O_4_/bentonite composite takes 30 min to reach equilibrium; the removal of Co(II), Cd(II), and Pb(II) by magnetic camel bone char takes 120 min to reach equilibrium [50]. The removal of Cd(II) by unmodified B_600_ takes 540 min to reach equilibrium (Figure 3a). In contrast, the composite adsorbent Cs-Fe_3_O_4_-B_600_ prepared in this experiment has certain advantages in achieving extremely fast adsorption of heavy metals.

The pseudo-first-order kinetics model (Equation (2)) and pseudo-second-order kinetics model (Equation (3)) were used to fit the obtained data. The relevant parameters obtained during the fitting process are shown in Table 1.

The pseudo-first-order equation is expressed as:

(2) $$\frac{dq_{t}}{dt}=k_{1}(q_{e}-q_{t})$$

The pseudo-second-order equation is expressed as:

(3) $$\frac{dq_{t}}{dt}=k_{1}{\left(q_{e}-q_{t}\right)}^{2}$$

where *q_e_* is the amount of Cd(II) adsorbed per unit mass of shell powder in adsorption equilibrium, mg·g^−1^; *q_t_* is the amount of Cd(II) absorbed per unit mass of shell powder at time *t*, mg·g^−1^; *t*: adsorption time (min); *k*_1_ is the rate constant, min^−1^; *k*_2_ is the pseudo-second-order rate constant, g·mg^−1^·min^−1^.

It can be seen from Table 1 that the adsorption of Cd(II) by Cs-Fe_3_O_4_-B_600_ is more in line with the pseudo-second-order kinetics model, and the correlation coefficient can reach 0.8989. The *q_e_*, cal (25.284 mg·g^−1^) obtained by the pseudo-second-order kinetics model fitting is closer to the experimental adsorption quantity *q_e_*, exp (25.134 mg·g^−1^). The pseudo-second-order kinetics model believes that the rate control step in the adsorption process is chemical adsorption, that is, in the adsorption process, through the covalent force and ion exchange between the adsorbent and the adsorbate, electrons are exchanged and shared, thereby forming chemical bonds [51,52].

#### 3.2.2. Adsorption Isotherm Characteristics

Cs-Fe_3_O_4_-B_600_ was used to adsorb Cd(II) with different initial concentrations. After the adsorption equilibrium was reached, the adsorption amount was calculated. The obtained data were fitted with the Langmuir adsorption isotherm model (Equation (4)) and Freundlich adsorption isotherm model (Equation (5)). The fitting results are shown in Figure 3c, and the relevant parameters in the fitting process are shown in Table 2.

(4) $$q_{e}=\frac{Q_{m}K_{L}C_{e}}{1+K_{L}C_{e}}$$

(5) $$q_{e}=K_{F}C_{e}{}^{\frac{1}{n}}$$

where *q_e_* is the equilibrium adsorption capacity, mg·g^−1^; *Q_m_* is the adsorption constant; *C_e_* is equilibrium concentration, mg·L^−1^; *K_L_* is adsorption parameters, L·mg^−1^; *K_F_* is adsorption capacity, mg·g^−1^(L·mg^−1^)^1/*n*^.

The correlation coefficient R^2^ of Langmuir equation fitting is 0.8653, which is higher than the correlation coefficient of the Freundlich equation of 0.7704. Therefore, compared with the Freundlich model, the adsorption of Cd(II) by Cs-Fe_3_O_4_-B_600_ is more in line with the Langmuir isotherm adsorption model. This model assumes that there is no interaction between the adsorbed molecules, that is, once Cd(II) occupies a certain adsorption site, the site will not undergo further adsorption. Therefore, it is assumed that the adsorption process is a single-layer adsorption and there is a saturated adsorption capacity. Cs-Fe_3_O_4_-B_600_ has abundant available adsorption sites on the surface at the beginning of the adsorption process. As the adsorption process continues, the number of available adsorption sites of Cs-Fe_3_O_4_-B_600_ decreases, so the adsorption amount slowly rises until it remains unchanged [53]. The saturated adsorption capacity *q_m_* obtained in this study is 64.310 mg·g^−1^, which is far greater than the saturated adsorption capacity of unmodified fish bone char B_600_ (37.799 mg·g^−1^, Table 2). Other studies have shown that the saturated adsorption capacity of Fe_3_O_4_/bentonite composite for Pb(II) can reach 81.5 mg·g^−1^, which is 10.7 mg·g^−1^ more than that of unmagnetic modified bentonite [54]. The saturated adsorption capacity of magnetic corn stover biochar for Cd(II) in water can reach 43.45 mg·g^−1^, which is greater than the 25.31 mg·g^−1^ of unmodified corn stover biochar [31]. Reza et al. [35] studied the removal of As(V) by magnetically modified bone char coated with chitosan. The results showed that the modified material is at 600 cm^−1^. Fe-O functional groups can be observed, the adsorption of arsenic is mainly monolayer adsorption, and the saturated adsorption capacity can reach 112,000 mg·g^−1^. In addition, *R_L_* is the separation factor. For the Langmuir adsorption isotherm model, it can be calculated by formula 6 to determine whether the reaction is favorable adsorption. In this experiment, *R_L_* < 1 indicates that the adsorption process is favorable adsorption.

(6) $$R_{L}=\frac{1}{1+K_{L}C_{e}}$$

#### 3.2.3. Influence of pH on Cd(II) Adsorption

**Figure 4:**
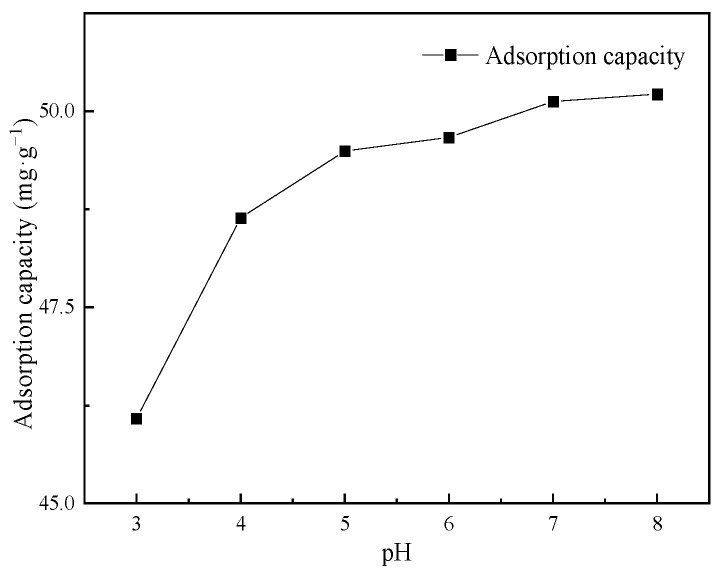
Effect of initial pH values on the adsorption of Cd(II) on Cs-Fe_3_O_4_-B_600_.

The relationship between the adsorption capacity of Cs-Fe_3_O_4_-B_600_ and the initial pH value is shown in Figure 4. Studies have shown that in the range of pH = 3–7 (when the acidity is low) Fe ions will not be generated in the system [31]; at the same time, at pH < 8, Cd mainly exists in the state of Cd(II). When the adsorbent dosage is 2 g·L^−1^ and the initial concentration is 100 mg·L^−1^, the adsorption capacity will increase significantly between pH = 3–5 and then slowly increase with the increase of pH. The adsorption capacity of Cs-Fe_3_O_4_-B_600_ varies from 46.08 to 48.64 mg·g^−1^ and 48.64 to 50.22 mg·g^−1^. The total increase in adsorption capacity is 4.14 mg·g^−1^, indicating that pH has a weak effect on the adsorption of Cd(II) on Cs-Fe_3_O_4_-B_600_. The trend of the relationship curve between the adsorption capacity of glutamic acid coupled chitosan modified activated carbon on Cd(II) and the initial pH value is consistent with this study [55].

Cs and B_600_ may combine with Fe_3_O_4_ through the surface hydroxyl group and the intermediate oxygen (-O-) in the carboxyl functional group. The involved process is shown in formula 7 and formula 8 [56]. The adsorption of Cd(II) by Cs-Fe_3_O_4_-B_600_ mainly includes ion exchange, surface complexation and precipitation of partially exposed to Cs-coated outer fish bone char, complexation of heavy metals with amino groups on Cs [57], and cations of composite materials specific adsorption, electrostatic adsorption. In addition, it may also include the ion exchange between isomorphic Cd(II) and Fe_3_O_4_ to produce CdFe_2_O_4_ [56]. When the pH of the solution is low, there is competitive adsorption of H^+^ and Cd(II); at the same time, the amino groups on the surface of Cs-Fe_3_O_4_-B_600_ are protonated in the presence of a large amount of H^+^ to form NH^3+^. The electrostatic repulsion between NH^3+^ and Cd(II) also makes the adsorption capacity low. With the increase of pH, the amino groups on the surface of chitosan are released, and the complexing ability of Cd(II) increases [58].

(7) $$R-OH+Fe_{3}O_{4}\to R-O-Fe_{3}O_{4}$$

(8) $$R-COOH+Fe_{3}O_{4}\to R-COO-Fe_{3}O_{4}$$

### 3.3. Characterization of Cs-Fe_3_O_4_-B_600_ before and after Adsorption

**Figure 5:**
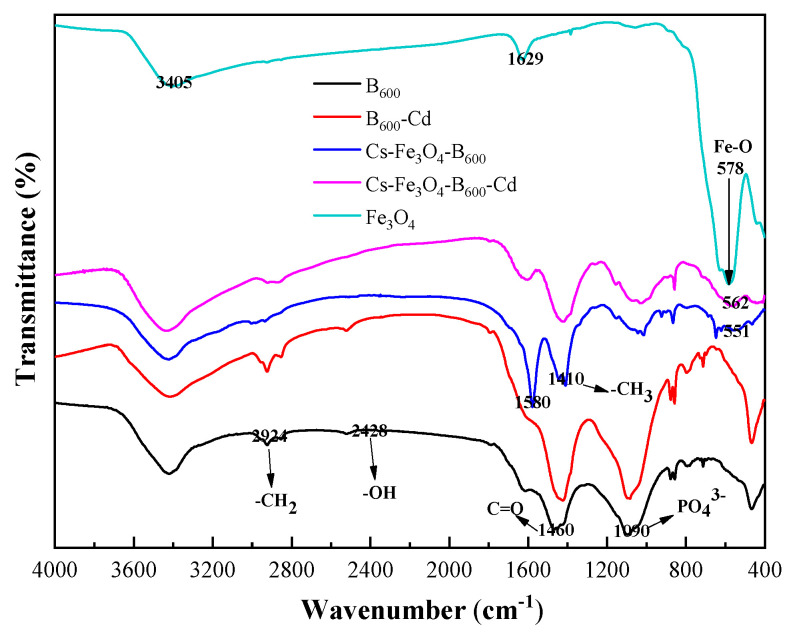
FTIR curves of Fe_3_O_4_, B_600_, B_600_-Cd, Cs-Fe_3_O_4_-B_600_, and Cs-Fe_3_O_4_-B_600_-Cd.

The FTIR spectra of Fe_3_O_4_, B_600_, B_600_-Cd, Cs-Fe_3_O_4_-B_600_, and Cs-Fe_3_O_4_-B_600_-Cd are shown in Figure 5. Fe_3_O_4_ has three prominent absorption peaks at 578, 1629, and 3405 cm^−1^. The peak at 550–580 cm^−1^ is considered to be the characteristic peak of Fe_3_O_4_, which is caused by the stretching vibration of Fe-O [59]. Compared with the FTIR spectrum of B_600_, Cs-Fe_3_O_4_-B_600_ and Cs-Fe_3_O_4_-B_600_-Cd have new absorption peaks at 551 and 562 cm^−1^, respectively, which are believed to be caused by Fe_3_O_4_ in the composite material. The prominent absorption peaks of B_600_ material exist at 1090, 1460, 2924, and 2428 cm^−1^, representing PO_4_^3−^, C=O, -CH_2_, and -OH, respectively. The coating effect of Cs may cause the weakening of PO_4_^3−^ strength in Cs-Fe_3_O_4_-B_600_. In addition, the absorption peaks of Cs-Fe_3_O_4_-B_600_ at 1410 and 1580 cm^−1^ are believed to be caused by the symmetrical variable-angle vibration of the -CH_3_ and amide II bands in Cs [60], which are based on Cs A slight red shift [59]. The appearance of Fe_3_O_4_ and Cs characteristic peaks in the Cs-Fe_3_O_4_-B_600_ material indicates that the material was successfully synthesized. Du et al. [30] used the co-precipitation method to prepare Fe_3_O_4_ magnetic biochar. The results showed that the infrared absorption peaks of C-H, C≡N, and Fe_3_O_4_ appeared after magnetic modification. The increase of hydroxyl, carboxyl, and other functional groups in the modification process enhances its ability to remove heavy metals through hydrogen bonding and ion exchange; the presence of aromatic functional groups can cause complexes with heavy metals; when the pH is high, heavy metals form hydroxyl complexes and magnetic materials can adsorb heavy metals through electrostatic interaction [61]. According to the FTIR results before and after Cs-Fe_3_O_4_-B_600_ adsorption of Cd(II), it can be seen that there are many band shifts, indicating that a variety of functional groups participate in the adsorption process of Cd(II). There are methyl or methylene absorption peaks at 1445 and 3034 cm^−1^, which move from 1445 to 1417 cm^−1^ after adsorption, and the carboxylic acid C=O stretching vibration peak at 1579 cm^−1^, which moves to 1608 cm^−1^. It shows that there are a large number of carboxyl groups and hydroxyl groups in the molecular structure of Cs-Fe_3_O_4_-B_600_. The hydroxyl groups and carboxyl groups can undergo ion exchange and complexation reactions with Cd(II). In this study, comparing the infrared spectra of Cs-Fe_3_O_4_-B_600_ and Cs-Fe_3_O_4_-B_600_-Cd, it can be found that the intensity of the absorption peak caused by the amide II band is weakened, which may be caused by the amino group complexing Cd(II). The research of Muzzarelli [57] showed that the amino group of Cs has a complexing effect on Cd(II).

**Figure 6:**
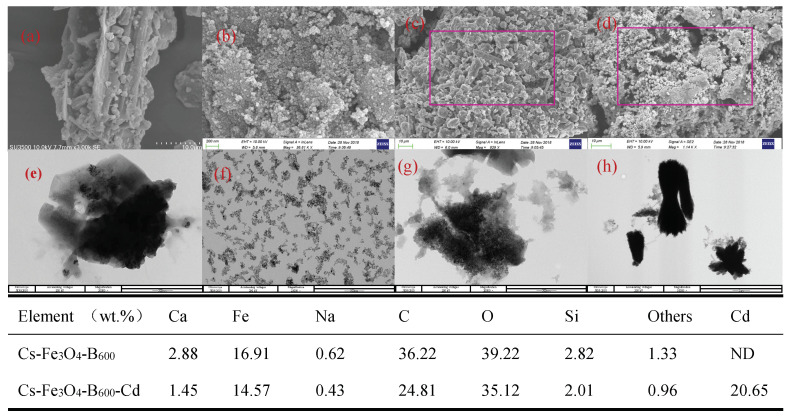
SEM and TEM images of B_600_ (**a**,**e**), Fe_3_O_4_ (**b**,**f**), Cs-Fe_3_O_4_-B_600_ (**c**,**g**), and Cs-Fe_3_O_4_-B_600_-Cd (**d**,**h**). Note: the table shows the element compositions of Cs-Fe_3_O_4_-B_600_ (**c**) and Cs-Fe_3_O_4_-B_600_-Cd (**d**).

The SEM images of Fe_3_O_4_, Cs-Fe_3_O_4_-B_600_, and Cs-Fe_3_O_4_-B_600_-Cd are presented in Figure 6. The Fe_3_O_4_ prepared in this experiment has spherical particles with a particle size of about 20–200 μm (Figure 6b), which is in contrast to Deng [62] and Peng [63] and other research conclusions are consistent. In addition, the surface of Fe_3_O_4_ particles is rough and coated with a large number of amorphous microspheres [64]. The TEM Figure 6f of Fe_3_O_4_ shows that most of the microspheres are intact. The SEM images of B_600_ (Figure 6a) show that there are cracks and gullies on the surface of B_600_, with a large number of mesopores and macropores. Comparing the SEM images of Cs-Fe_3_O_4_-B_600_ (Figure 6c), it can be found that Cs wrapped B_600_ and Fe_3_O_4_, blocked the macropores of B_600_, and formed a rough outer surface structure. This conclusion is consistent with the BJH pore size distribution of Cs-Fe_3_O_4_-B_600_. Figure 6g shows that the spherical particles are coated. Figure 6d can clearly observe the spherical Fe_3_O_4_ particles attached to the bone char in Cs-Fe_3_O_4_-B_600_-Cd, that is, the spindle-shaped Cd(II) attached to it. The transmission electron microscope can also observe the obvious Cd spindle structure, showing that Cd(II) is adsorbed on it. The EDS analysis of Cs-Fe_3_O_4_-B_600_-Cd showed that after adsorption, Cs-Fe_3_O_4_-B_600_ detected 20.65 wt.% Cd, which confirmed the consistency of SEM and TEM analysis results.

## 4. Conclusions

Cs-Fe_3_O_4_-B_600_ was successfully fabricated, modified with chitosan and Fe_3_O_4_ based on B_600_. The kinetic data of Cs-Fe_3_O_4_-B_600_ are in the best agreement with the pseudo-second-order model, and the maximum Cd^2+^ adsorption capacity by Cs-Fe_3_O_4_-B_600_ is 25.284 mg·g^−1^ in solution when the initial Cd^2+^ concentration is 150 mg·L^−1^. Adsorption isotherms are in better accordance with Langmuir models. Thermodynamic analysis explained that the adsorption process, which was monolayer adsorption, was a favorable adsorption. The saturated adsorption capacity of Cs-Fe_3_O_4_-B_600_ for Cd(II) increased from 37.799 mg·g^−1^ before unmodified to 64.31 mg·g^−1^. Cs-Fe_3_O_4_-B_600_ was analyzed using different techniques (SEM-EDS, TEM, BET, FTIR, and XRD). The resulting Cs-Fe_3_O_4_-B_600_ composites manifested tremendous physicochemical properties such as the appearance of amino functional groups, larger specific surface area, and larger pore volume. Cd(II) successfully adsorbed onto Cs-Fe_3_O_4_-B_600_ because of the amino complexation, ion exchange, precipitation, cation-specific adsorption, and electrostatic adsorption. In conclusion, applying Cs-Fe_3_O_4_-B_600_ could be a low-cost, fast, and efficient solution for the removal of Cd(II) in water.

## Figures and Tables

**Table 1:** Pseudo-first-order and pseudo-second-order kinetics models for Cd(II) adsorption on B_600_ and Cs-Fe_3_O_4_-B_600_.

Materials	C_0_ (mg·L^−1^)	*q_e_*, exp (mg·g^−1^)	Pseudo-First-Order Kinetics Model			Pseudo-Second-Order Kinetics Model		
			*q_e_*, cal (mg·g^−1^)	*k*_1_ (1·min^−1^)	R^2^	*q_e_*, cal (mg·g^−1^)	*k*_2_ (g·mg^−1^·min^−1^)	R^2^
B_600_	150	19.487	20.792	0.0034	0.9385	26.696	0.00001	0.9081
Cs-Fe_3_O_4_-B_600_	150	25.134	25.284	0.4825	0.8989	25.871	0.0302	0.8305

**Table 2:** Langmuir and Freundlich isotherm models for Cd(II) adsorption on Cs-Fe_3_O_4_-B_600_ and B_600_.

Adsorption Isotherm Model	Parameter	Adsorbent	Adsorbent
		Cs-Fe_3_O_4_-B_600_	B_600_
Langmuir	*q_m_* (mg·g^−1^)	64.310	37.799
	*K_L_* (L·mg^−1^)	2.0890	0.0591
	R^2^	0.8653	0.9892
Freundlich	*K_F_* (mg·g^−1^(L·g^−1^)^1/*n*^)	39.804	7.4522
	*n*	8.9967	3.1546
	R^2^	0.7704	0.9362

## Data Availability

The data presented in this study are available on reasonable request from the corresponding author. The data are not publicly available due to privacy or ethical considerations.

## Supplementary Materials

The following supporting information can be downloaded at: https://www.mdpi.com/article/10.3390/ijerph19031260/s1, Figure S1: XRD pattern of Fe_3_O_4_.
