# Supplementary figures and images for: Adsorption Performance of Cd(II) by Chitosan-Fe3O4-Modified Fish Bone Char
Source: Int J Environ Res Public Health. 2022 Jan 23;19(3):1260. doi: 10.3390/ijerph19031260 (PMC8834754; doi:10.3390/ijerph19031260)

## Supplementary Material

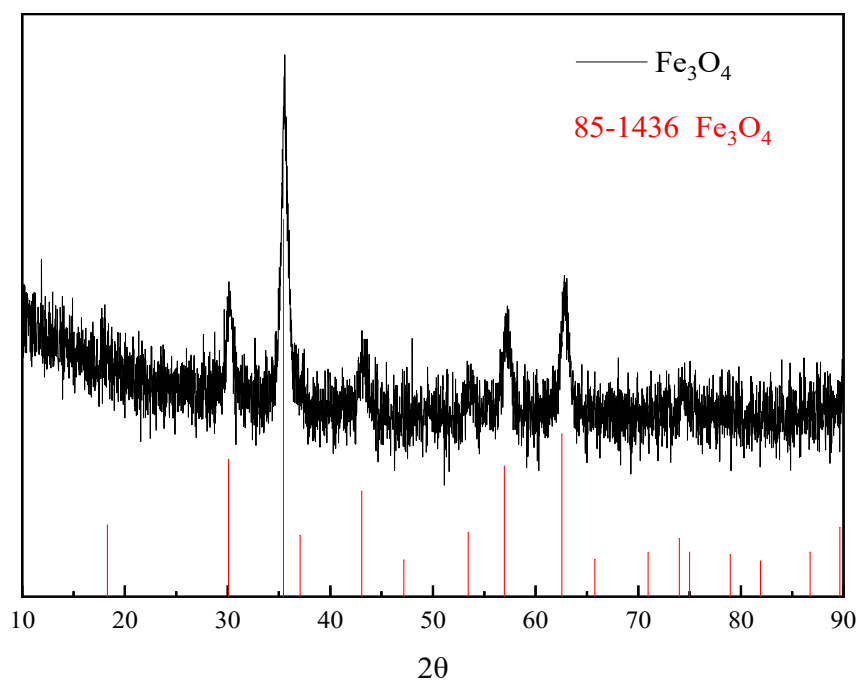

**Figure S1.** XRD pattern of  $\text{Fe}_3\text{O}_4$ .

Supplement: Supplementary file 1 [file ijerph-19-01260-s001.zip › ijerph-1528617-supplementary.pdf]
